# Proteasome inhibitors prevent bi-directional HER2/estrogen-receptor cross-talk leading to cell death in endocrine and lapatinib-resistant HER2+/ER+ breast cancer cells

**DOI:** 10.18632/oncotarget.20261

**Published:** 2017-08-14

**Authors:** Sonja Thaler, Marcus Schmidt, Sven Roβwag, Gitta Thiede, Arno Schad, Jonathan P Sleeman

**Affiliations:** ^1^ Centre for Biomedicine and Medical Technology Mannheim (CBTM), Medical Faculty Mannheim, University of Heidelberg, Mannheim, Germany; ^2^ Department of Obstetrics and Gynecology, University Medical Center, Johannes Gutenberg University, Mainz, Germany; ^3^ Institute of Pathology, University Medical Center, Johannes Gutenberg University, Mainz, Germany; ^4^ KIT Campus Nord, Institute for Toxicology and Genetics, Karlsruhe, Germany

**Keywords:** proteasome inhibitors, ER+/HER2+ breast cancer, breast cancer therapy

## Abstract

Amplification and/or overexpression of the human epidermal growth factor 2 (HER2) oncogene occurs in about 13–15% of invasive breast cancer and triggers breast cancer cell proliferation, survival and metastatic progression. Around half of all breast cancers with HER2 overexpression co-express hormone receptors (HR) such as those for estrogen and progesterone. Aberrant signaling through HER2 and other members of the HER-family mediates endocrine-resistance in estrogen receptor alpha (ERα) positive breast cancer. On the other hand, ERα co-expression has been shown to attenuate the efficiency of anti-HER2 therapies. These findings indicate that HER2 and ERα synergize to escape from both anti-ERα and anti-HER2-targeted therapies. Rationally designed clinical trials that combine endocrine therapy with anti-HER2 agents to interfere with HER2/ERα cross-talk have been conducted. However, the outcome of these trials suggests that novel therapeutic approaches are needed to further improve inhibition of HER2 and other HER-family members in conjunction with a more efficient ERα blockade. Here, we demonstrate that carfilzomib and bortezomib stabilize the HER2-specific protein tyrosine phosphatase BDP1 leading to decreased HER2 autophosphorylation, reduced HER2 activity and subsequently attenuated activation of the PI3K/Akt-pathway, together with blockade of ERα expression. We further observed that proteasome inhibitors (PIs) reverse autophosphorylation and thereby inhibit the activity of constitutively active mutant HER2. We also demonstrate that PIs cause cell death in lapatinib and endocrine-resistant HER2+/ER+ breast cancer cells. These findings suggest that PIs might have the potential to improve the management of HER2+/ER+ breast cancer patients by efficiently disrupting the bi-directional HER2/ERα cross-talk.

## INTRODUCTION

The human epidermal growth factor receptor 2 (HER2) oncogene is amplified and/or overexpressed in around 15% of invasive breast cancer. Breast cancers with HER2 gene amplification and/or HER2-overexpression are commonly characterized by poor clinical outcome, aggressive behaviour, higher recurrence rate and increased mortality [1, 2]. Although HER2-targeted therapies in combination with chemotherapy have significantly improved the treatment and survival of HER2+ breast cancer patients [3–10], in clinical practice HER2+ breast cancer patients are not homogeneous in terms of disease progression, prognosis and response to therapeutic regimens [11]. In particular, co-expression of hormone receptors (HR) and especially ER alpha (ERα) appears to be a significant predictor of differential sensitivity to anti-HER2 and chemotherapy based therapies [12]. Data from several neoadjuvant studies suggest that the response to therapy with anti-HER2 agents markedly depends on HR status and ERα expression in particular [8, 9, 12, 13, 14].

*In vitro* studies based on HER2+ breast cancer cell lines with either intrinsic or acquired resistance to trastuzumab, lapatinib or both trastuzumab and lapatinib have been performed to determine the role of ERα in the onset of resistance to HER2-targeted therapies [15]. The results of these experiments showed that under sustained HER2 inhibition, ERα can rescue HER2+/ER+ cells, and that the dynamic switch between HER2 and ERα activity plays a central role in determining resistance to lapatinib-containing treatment regimens [15]. In clinical practice, increased ERα activity has also been reported in patients with HER2+/ERα+ metastatic breast cancer [16, 17]. Thus, these observations indicate that either ERα or HER2 can function as a major promoter of proliferation and survival in HER2+/ER+ breast cancer cells.

Upregulated expression of ERα serves as a survival mechanism upon permanent HER2 inhibition, while increased signaling through HER2 and/or other members of the HER-family has been shown to mediate resistance to endocrine therapies in ERα+ breast cancer cells [18, 19]. Sustained activation of the PI3K/Akt and the Ras/MAPK pathways through these and other receptors such as IGF-R1 is considered to be the most important mechanism that leads to endocrine resistance [18, 19]. Phosphorylation of ERα and its co-activators by these pathways was found to lead to enhanced genomic ERα activity and increased expression of ERα-target genes, even in the absence of estrogen or in the presence of tamoxifen [20–22]. Phosphorylation of co-repressors causes their inactivation and export out of the nucleus, thereby increasing expression of ERα-target genes [23, 24].

Two further mechanisms illustrate how ERα can influence HER2 expression to determine tamoxifen resistance. First, it was shown that in the presence of the transcription factor PAX2 estrogen-ERα and tamoxifen-ERα complexes directly repress HER2 transcription. Thus, inhibition of PAX2 causes tamoxifen resistance through ERα-mediated transcriptional up-regulation of HER2 [25]. Second, the interaction between the co-activator HOXB7 and ERα leads to tamoxifen resistance through overexpression of the ERα-target genes HER2 and Myc [26]. Thus, both reports indicate that HER2 is an ERα-target gene and that transient up-regulation of HER2 expression by ERα can cause endocrine resistance [25, 26].

In conclusion, these observations highlight the importance of dual inhibition of both HER2 and ERα to achieve the most efficient antitumor activity in HER2+/ER+ breast cancer. Clinical studies using endocrine therapy combined with HER2-targeting agents have already been conducted in an attempt to block HER2 and ERα cross-talk [27–30]. However, these trials showed only a modest activity of the dual blockade of both ERα and HER2. In the more recently reported PERTAIN trial advanced HR+/HER2+ breast cancer patients were treated with an aromatase inhibitor (AI) and trastuzumab either with or without pertuzumab treatment [31]. This study has demonstrated that patients receiving additional pertuzumab had an increased progression-free survival (PFS) [31], confirming that effective suppression of both HER2 and ERα are crucial to improve HER2+/HR+ breast cancer treatment. Nevertheless, further novel therapeutic strategies that more efficiently inhibit both HER2 and ERα are required. Furthermore, the observation that ERα-mediated transient up-regulation of HER2 leads to endocrine resistance suggests that therapeutic regimens leading to dual blockade of ERα and HER2 even in ER+ breast cancers without HER2 amplification or primarily HER2 overexpression might be therapeutically relevant.

In a previous report we have shown in ER+ breast cancer cell lines that the first generation proteasome inhibitor (PI) bortezomib decreased expression of ERα and HER2 and inhibited signalling pathways responsible for induction of endocrine resistance [32]. These observations led us to suggest that PIs might be a possible treatment option for endocrine therapy resistant ERα+ breast cancers and prompted us to investigate further whether PIs can target ERα+/HER2-amplified breast cancer cells, as well as the mechanisms through which they act. To determine whether different PIs act through equivalent mechanisms within cells, we performed experiments with bortezomib and the second generation PI carfilzomib in parallel. We found that carfilzomib and bortezomib markedly inhibit bi-directional HER2/ERα signaling pathways in HER2+/ER+ breast cancer cell lines. Both PIs suppress ERα expression, inhibit HER2, and subsequently suppress the downstream pathway PI3K/Akt that is a major executor of endocrine resistance. Furthermore, these PIs stabilized the HER2-specific tyrosine phosphatase BDP1, and were thereby even able to suppress the activity of a constitutively active HER2 variant that is resistant to trastuzumab and lapatinib. These findings indicate that PIs substantially disrupt the cross-talk between HER2 and ERα signaling pathways through mechanisms other than those activated by the therapeutic regimens currently used to treat HER2+/ER+ breast cancer. While clinical application of bortezomib has often been limited due to high toxicity clinical trials performed with carfilzomib showed a more favourable side effect profile [33], suggesting that carfilzomib might have the potential to improve treatment of HER2+/ER+ breast cancer patients. Furthermore, these data suggest that carfilzomib could also be a treatment option for patients with endocrine resistant HER2-/ER+ breast cancer, or for patients with mutated HER2. An early phase clinical trial of carfilzomib would therefore be useful to test its value in the treatment of advanced breast cancer.

## RESULTS

### Carfilzomib leads to down-regulation of ERα expression and induces cell death in ER+ breast cancer cells

Recently, we reported that the first generation PI bortezomib inhibits ERα-expression and disrupts signaling pathways that mediate resistance to commonly used treatment regimens that target ERα [32]. These findings led us to hypothesize that PIs might have the potential to be a possible treatment option for ERα+ breast cancers, including those resistant to endocrine therapy. In the present study we have investigated whether the second generation PI carfilzomib exerts inhibitory effects on ERα+ breast cancer cells equivalent to those of bortezomib, and whether both drugs can target endocrine therapy-resistant ERα+/HER2-amplified breast cancer cells.

**Figure 1:**
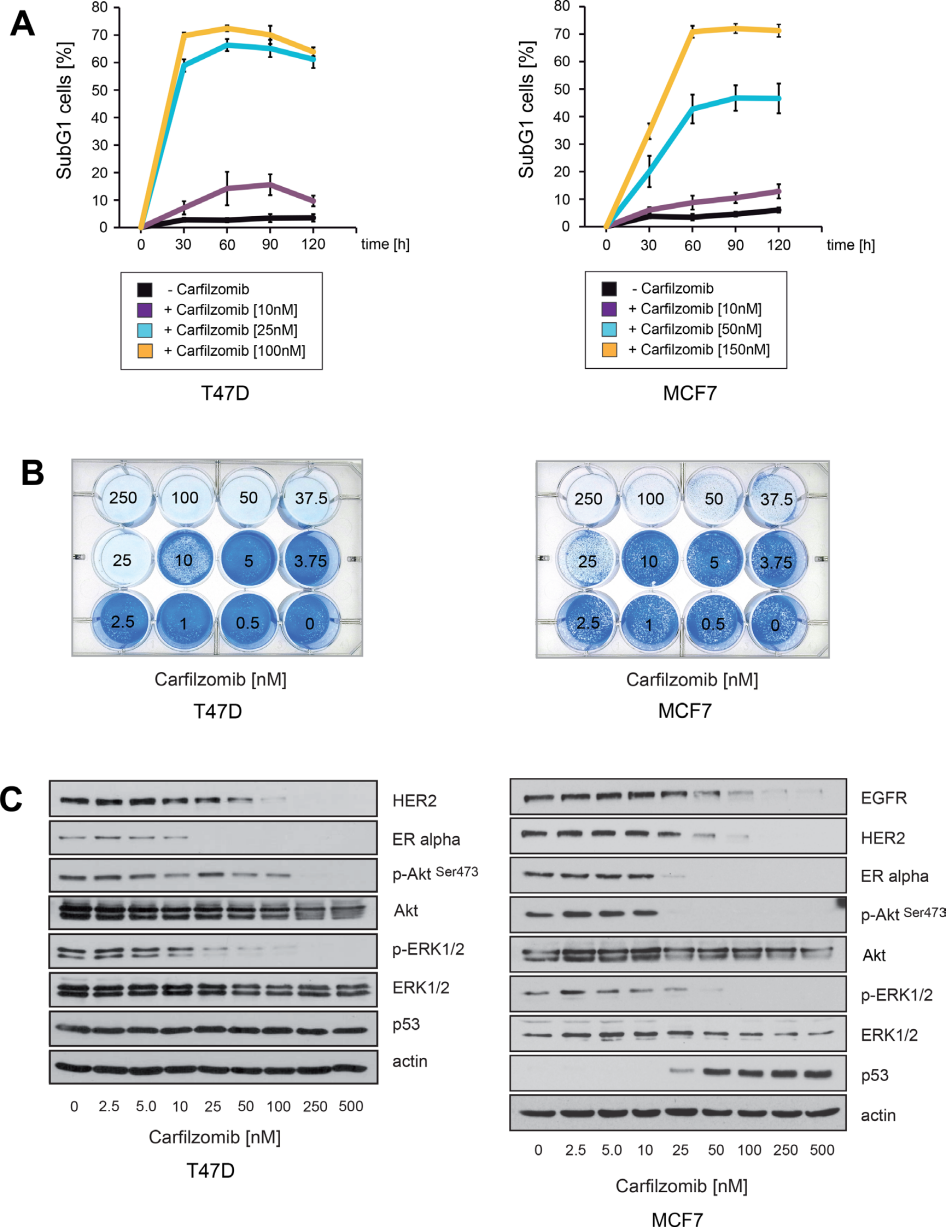
Carfilzomib down-regulates ERα expression and induces cell death in ER+ breast cancer cells (**A**) Carfilzomib induces cell death in a concentration- and time dependent manner. Cells were cultured in the presence or absence of the indicated carfilzomib concentrations. The percentage of SubG1 cells was evaluated at different time points using propidium iodide staining and flow cytometry. Mean values ± s.d. of three independent experiments are presented. (**B**) Equal numbers of T47D and MCF7 cells were seeded on 12-well culture plates and treated with the indicated carfilzomib concentrations. After 10 days cells were fixed and stained. (**C**) T47D and MCF7 cells were cultured with the indicated carfilzomib concentrations for 32 or 36 hours, respectively. Western blots of protein lysates were probed with the indicated antibodies. β-actin served as loading control.

Induction of cell death upon carfilzomib treatment was assessed by quantification of the SubG1 DNA content in treated cells and through the use of colony-forming assays (Figure 1A, 1B). Notably, after 10 days no surviving cells could be detected when MCF7 cells were cultured in the presence of 50–100 nM carfilzomib nor when T47D cells were treated with 25 nM carfilzomib (Figure 1B). Immunoblotting demonstrated that carfilzomib causes decreased expression of ERα and decreased levels of HER2, as well as inhibition of Akt and MAPK, as indicated by reduced amounts of p-Akt^Ser473^ and p-ERK1/2 in both cell lines (Figure 1C). Collectively, these results indicate that carfilzomib has similar effects on ERα+ breast cancer cells as those previously reported for bortezomib [32].

### Carfilzomib and bortezomib inhibit HER2 autophosphorylation, block ERα expression and cause cell death in ER+/HER2-amplified breast cancer cells

**Figure 2:**
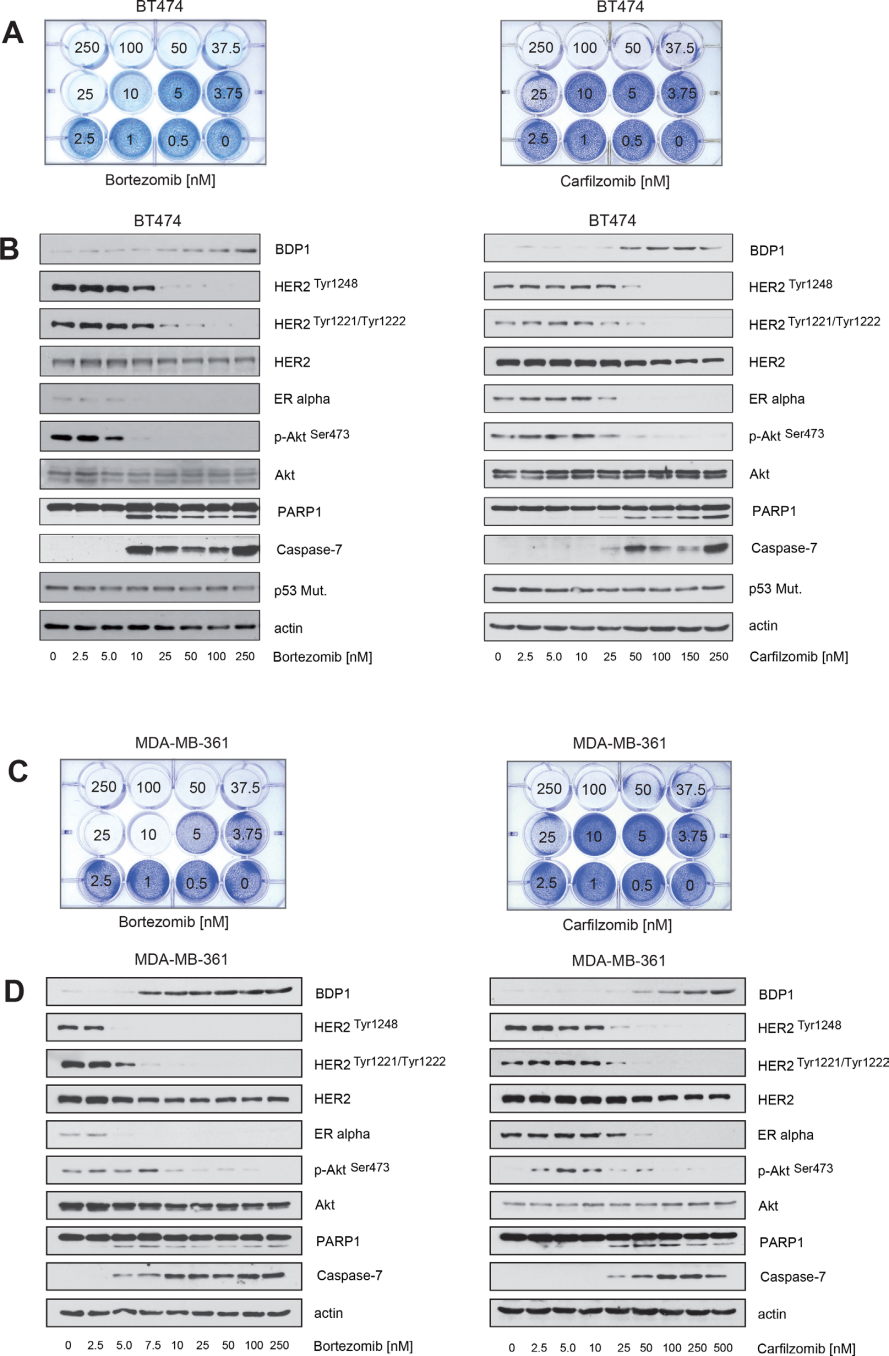
Carfilzomib and bortezomib inhibit HER2 autophosphorylation, block ERα expression and cause cell death in ER+/HER2-amplified breast cancer cells (**A**–**D**) Carfilzomib and bortezomib induce cell death in a concentration-dependent manner. (A) and (C) Equal numbers of BT474 or MDA-MB-361 cells were seeded on 12-well culture plates and treated with the indicated carfilzomib or bortezomib concentrations. After 10 days cells were fixed and stained. (B) and (D) BT474 and MDA-MB-361 cells were cultured in the presence of the indicated carfilzomib and bortezomib concentrations for 32 hours. Western blots of protein lysates were probed with the indicated antibodies.

To determine whether PIs can act against ERα+ breast cancer cells that have intrinsic resistance to endocrine therapy regimens, the ER+/HER2+ breast cancer cell lines BT474 and MDA-MB-361 were cultured in the presence of various carfilzomib or bortezomib concentrations. Induction of cell death upon carfilzomib and bortezomib treatment was assessed using colony-forming assays (Figure 2A, 2C). Apoptosis was monitored by immunoblotting and detection of caspase7 and Poly(ADP-ribose)polymerase-1 (PARP1) cleavage (Figure 2B, 2D). After 9 days, no surviving cells could be detected when BT474 or MDA-MB-361 cells were cultured in the presence of carfilzomib concentrations higher than 25–50 nM (Figure 2A, 2C, right panels) or with bortezomib concentrations higher than 5–10 nM (Figure 2A, 2C, left panels).

Both carfilzomib and bortezomib decreased expression of ERα, suppressed HER2 autophosphorylation, and inhibited Akt, as indicated by decreased levels of p-HER2^Tyr1248^, p-HER2^Tyr1221/1222^ and p-Akt^Ser473^ (Figure 2B, 2D). Caspase and PARP1 cleavage correlate with reduced HER2 phosphorylation and reduced ERα levels (Figure 2B, 2D).

Activation of HER2 triggers autophosphorylation of specific tyrosine residues within its cytoplasmic domain, subsequently leading to activation of intracellular signaling pathways such as the Ras/MAPK and the PI3K/Akt pathways [34]. Protein tyrosine phosphatases are important key regulators of HER2 activity [35, 36], including the PEST-type protein-tyrosine phosphatase BDP1 that inhibits ligand-induced activation of HER2 by decreasing HER2 phosphorylation [36, 37]. PEST-type proteins are rapidly degraded by the ubiquitin/proteasome system [38]. Based on this information, we hypothesized that PIs might increase the half-life of BDP1 and other PEST-type proteins such as PTPN12 [39] and PTPN13 [35], thereby decreasing the phosphorylation state and reversing the activation of HER2. Accordingly, using immunoblotting we found that increased levels of BDP1 were observed in the presence of increasing carfilzomib and bortezomib concentrations and that BDP1 levels and the phosphorylation status of HER2 were almost inversely related (Figure 2B, 2D).

**Figure 3:**
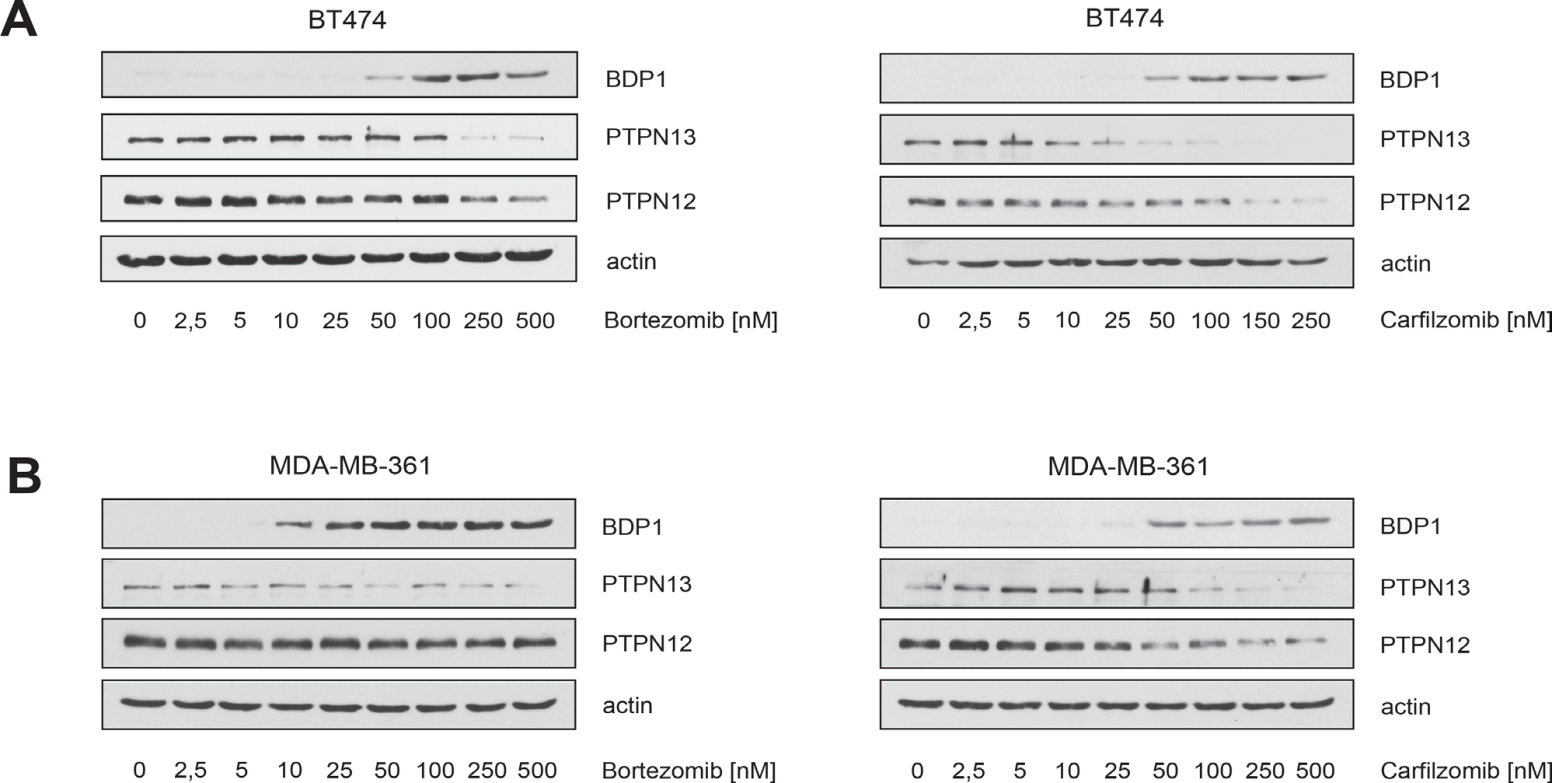
Carfilzomib and bortezomib increase the levels of the PEST type protein-tyrosine phosphatase BDP1 (PTPN18) but not PTPN12 and PTPN13 (**A**) BT474 and (**B**) MDA-MB-361 cells were cultured in the presence of the indicated carfilzomib (right panels) and bortezomib (left panels) concentrations for 32 hours. Western blots of protein lysates were probed with the indicated antibodies.

Notably, only BDP1 but not PTPN12 or PTPN13 levels were increased within BT474 and MDA-MB-361 cells in the presence of PIs (Figure 3). These findings are consistent with the notion that decreased HER2 phosphorylation upon PI treatment may be mediated by BDP1.

### Carfilzomib and bortezomib-mediated inhibition of HER2 phosphorylation, blockade of ERα expression and increased levels of BDP1 correlate temporally with induction of apoptotic cell death

**Figure 4:**
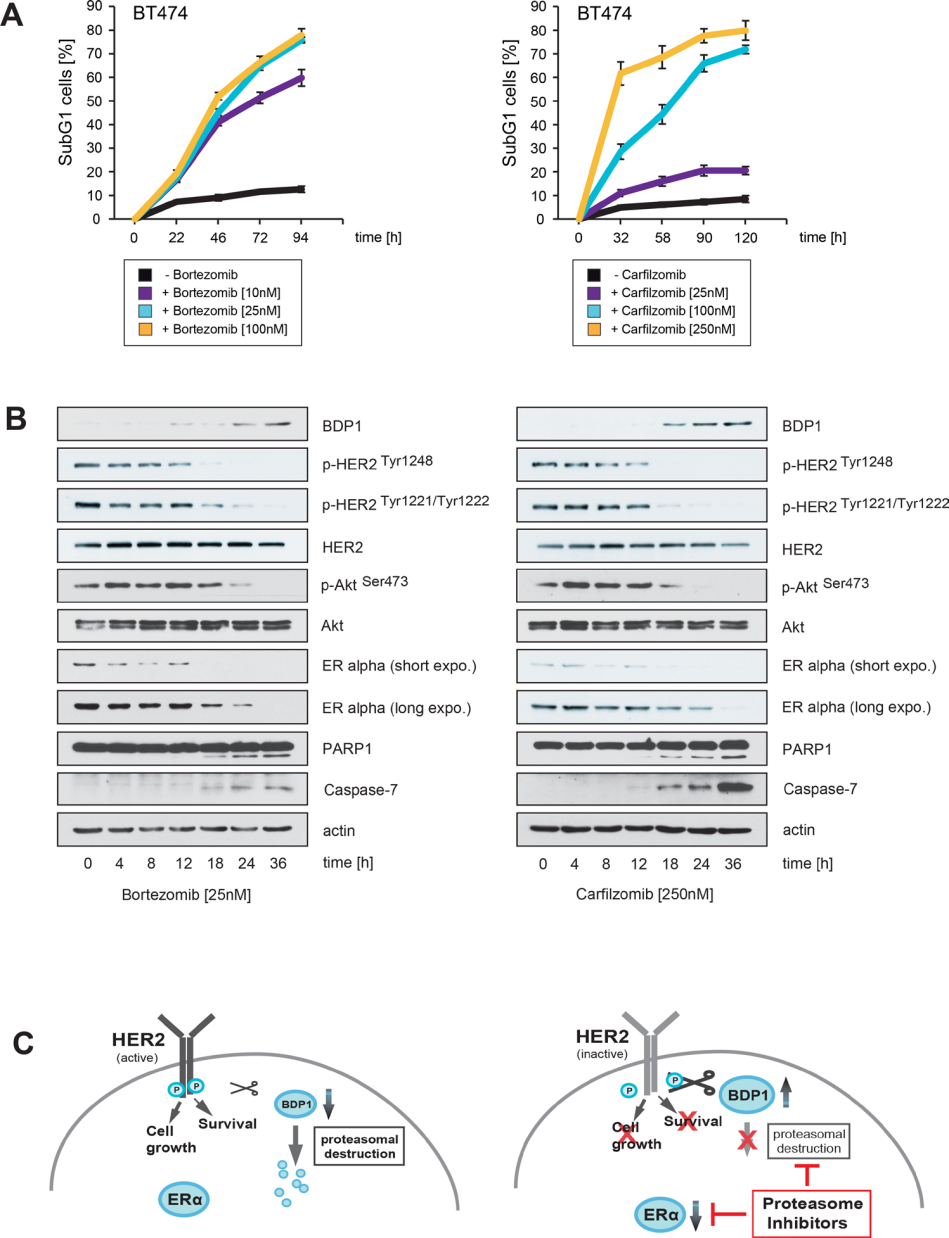
Carfilzomib and bortezomib-mediated inhibition of HER2 autophosphorylation, blockade of ERα expression and increase in BDP1 levels correlates temporally with induction of apoptotic cell death (**A**) Carfilzomib and bortezomib induce cell death in a time-dependent manner. BT474 cells were cultured in the presence or absence of the indicated carfilzomib or bortezomib concentrations. The percentage of SubG1 cells was evaluated at different time points using propidium iodide staining and flow cytometry. Mean values ± s.d. of three independent experiments are presented. (**B**) BT474 cells were cultured in the presence of 250 nM carfilzomib or 25 nM bortezomib. Cells were harvested at the indicated time points. Western blots of protein lysates were probed with the indicated antibodies. (**C**) Schematic model of a possible molecular network regulated by both proteasome inhibitors, based on the results of the Western blots and the literature. Continued activation of HER2 has a causal role in tumorigenesis and leads to tumor progression [1, 2]. Activation of HER2 triggers autophosphorylation of specific tyrosine residues within its cytoplasmic domain, consequently activating different intracellular signaling pathways such as Ras-MAPK and the PI3K/Akt axis [34] which determine cell growth, cell survival and therapy resistance to endocrine therapies. Different protein tyrosine phosphatases regulate phosphorylation of the HER2 signaling domain and are therefore important key regulators of HER2 activity [35, 36]. The PEST-type protein-tyrosine phosphatase BDP1 inhibits ligand-induced activation of HER2 [36, 37]. PEST sequences cause accelerated degradation by the proteasome/ubiquitin system [38], and PIs therefore increase the amount of BDP1 through blocking its proteasomal destruction. This in turn leads to accelarated dephosporylation and thereby inactivation of HER2 and its downstream target signaling pathways. Aberrant signaling through HER2 and other members of the HER family mediates endocrine resistance in ER+ positive breast cancer. On the other hand ERα co-expression with HER2 attenuates the efficiency of anti-HER2-targeted therapies. These findings indicate that HER2 and ERα act in concert to allow breast cancer cells to escape from both anti-ERα and anti-HER2-targeted therapies. Besides inhibiting HER2, PIs also suppress ERα protein expression.

The results in Figure 2 were obtained after cells had been incubated with PIs for more then 30 hours (Figure 2B, 2D) or even several days (Figure 2A, 2C). However, quantification of the SubG1 DNA content in BT474 cells revealed that cell death in response to PI treatment is induced at earlier time points (Figure 4A). We therefore investigated whether induction of apoptotic cell death as measured by caspase and PARP1 cleavage correlates temporally with decreased HER2 phosphorylation, BDP1 up-regulation and decreased expression of ERα. To this end, BT474 cells were treated with either 25 nM bortezomib or 250 nM carfilzomib, then cells were harvested at different time points and analysed by immunoblotting (Figure 4B).

Notably, the experiments showed that increased BDP1 levels, decreased HER2 phosphorylation and reduced ERα expression occured at earlier time points after PI treatment, concomitantly with caspase and PARP1 cleavage. Together these results suggest that PIs efficiently inhibit ERα/HER2 cross-talk pathways through blocking ERα expression, as well as through inhibiting HER2 activation via stabilization of BDP1, which leads to the death of ER+/HER2+ breast cancer cells (Figure 4C).

### Knockdown of BDP1 attenuates PI-mediated inactivation of HER2 and its downstream target Akt

To determine whether BDP1 is functionally involved in PI-mediated suppression of HER2 autophosphorylation and inhibition of Akt, stable knockdowns of BDP1 were performed.

**Figure 5:**
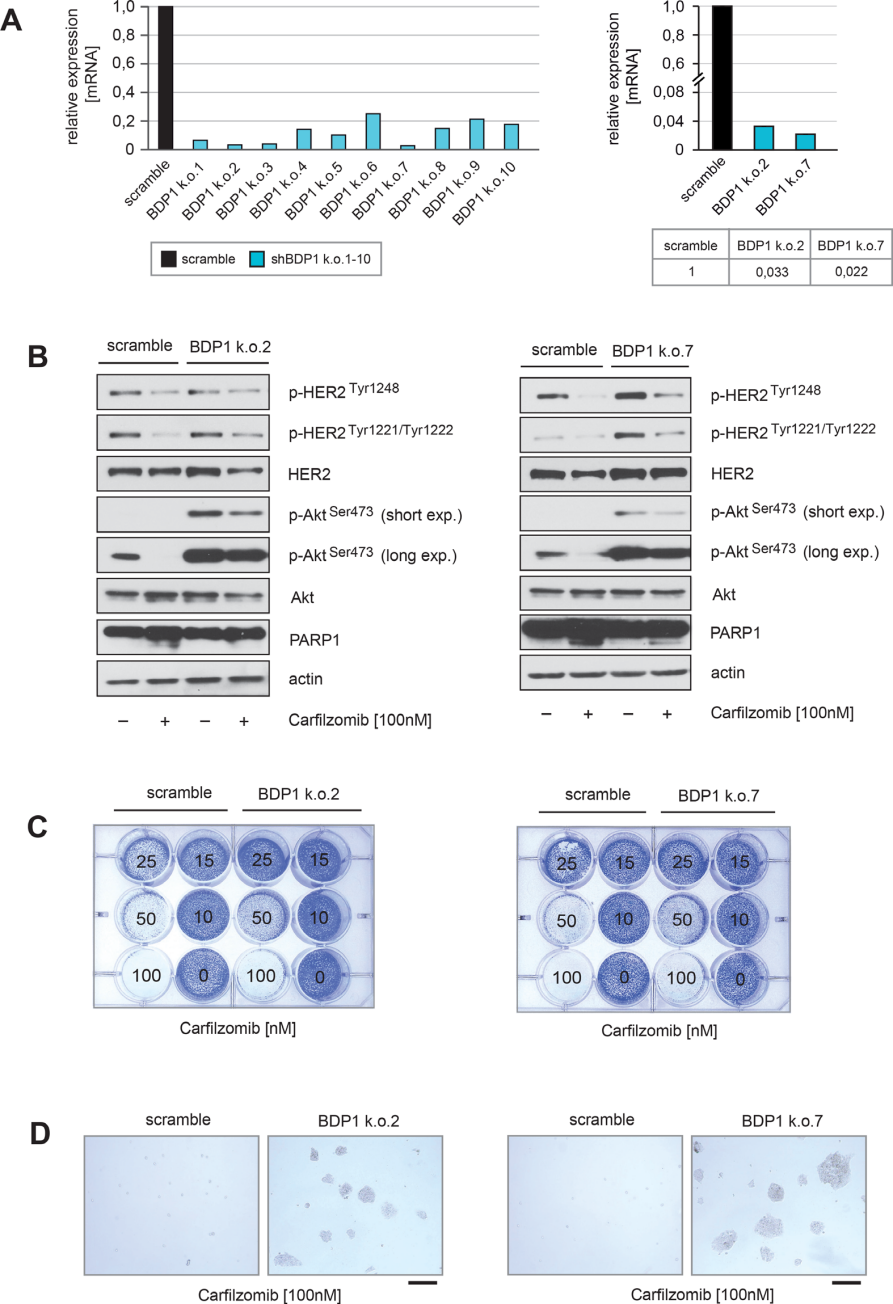
Knockdown of BDP1 attenuates PI-mediated inactivation of HER2 and its downstream target Akt (**A**) Stable knockdown of BDP1 within BT474 cells was performed by using lentiviral transfer of non-targeted or ten different targeted shRNAs against BDP1. After transduction reduced expression of BDP1 in ten BT474 BDP1 k.o. cells was evidenced by qPCR (left panel). The two shRNAs (k.o.2 and k.o.7) with the highest potential to decrease BDP1 expression in BT474 cells at passage 2 of puromycin selection were used to quantify the degree of knockdown (right panel). (**B**) BT474 cells either transduced with non-targeted or BT474 k.o.2 and k.o.7 from passage 2 of puromycin selection were cultured in the presence or absence of carfilzomib for 28 hours. Western blots of protein lysates were probed with the indicated antibodies. (**C**) Equal numbers of BDP1 k.o.2, BDP1 k.o.7 and negative control scramble BT474 cells were seeded on 12-well culture plates and treated with the indicated carfilzomib concentrations (right panels). After 9 days cells were fixed and stained. (**D**) Macroscopic photos are shown of the fixed cell colonies for BT474 cells either untreated or treated with the indicated carfilzomib concentration. Bar 100 μm.

The efficiency of the shRNAs to inhibit BDP1 expression within BT474 cells was measured by quantitative PCR (Figure 5A, left panel). The two most effective shRNAs reduced BDP1 mRNA transcripts approximately 30–45 fold after two passages under selection (Figure 5A, right panel). BT474 cells stably transduced with either BDP1 shRNA k.o.2, k.o.7 or with non-targeted scrambled shRNA were then used for functional analysis. Immunoblotting revealed that both BDP1 knock down cell lines displayed much stronger activation of Akt, as indicated by higher levels of p-Akt^Ser473^ in comparison with their scrambled shRNA control counterparts (Figure 5B). BDP1 k.o.7 cells also displayed increased endogenous levels of p-HER2^Tyr1248^ and p-HER2^Tyr1221/1222^ in comparison to k.o.2 cells and controls. The HER2 downstream target Akt was strongly activated in both BDP1 knockdown cells, possibly because BDP1 might increase phosphorylation of other HER2 phosphorylation sites that are important for activation of Akt such as p-HER2^Tyr1112^ and p-HER2^Tyr1196^ [37] or because BDP1 affects the activation status of Akt directly by mechanisms other than HER2 inhibition.

Immunoblotting also revealed that after BDP1 knockdown, the ability of carfilzomib to decrease HER2 autophosphorylation and to inhibit Akt activation was attenuated, as indicated by higher levels of p-HER2^Tyr1248^, p-HER2^Tyr1221/1222^ and p-Akt^Ser473^ (Figure 5B). In the presence of carfilzomib both BDP1 knockout cell lines showed decreased amounts of cleaved PARP1 (Figure 5B) indicating an insensitivity to PI compared to controls. Furthermore, only BDP1 knockout cells formed colonies in the presence of carfilzomib concentrations higher than 50 nM (Figure 5C, 5D).

These findings indicate that knockdown of BDP1 is functionally involved in PI-mediated inhibition of HER2 and Akt, thereby making BT474 cells less sensitive to PI-induced cell death.

### Establishment of fulvestrant-resistant ERα-positive breast cancer cells through expression of a constitutively active HER2 mutant that is resistant to trastuzumab and lapatinib

**Figure 6:**
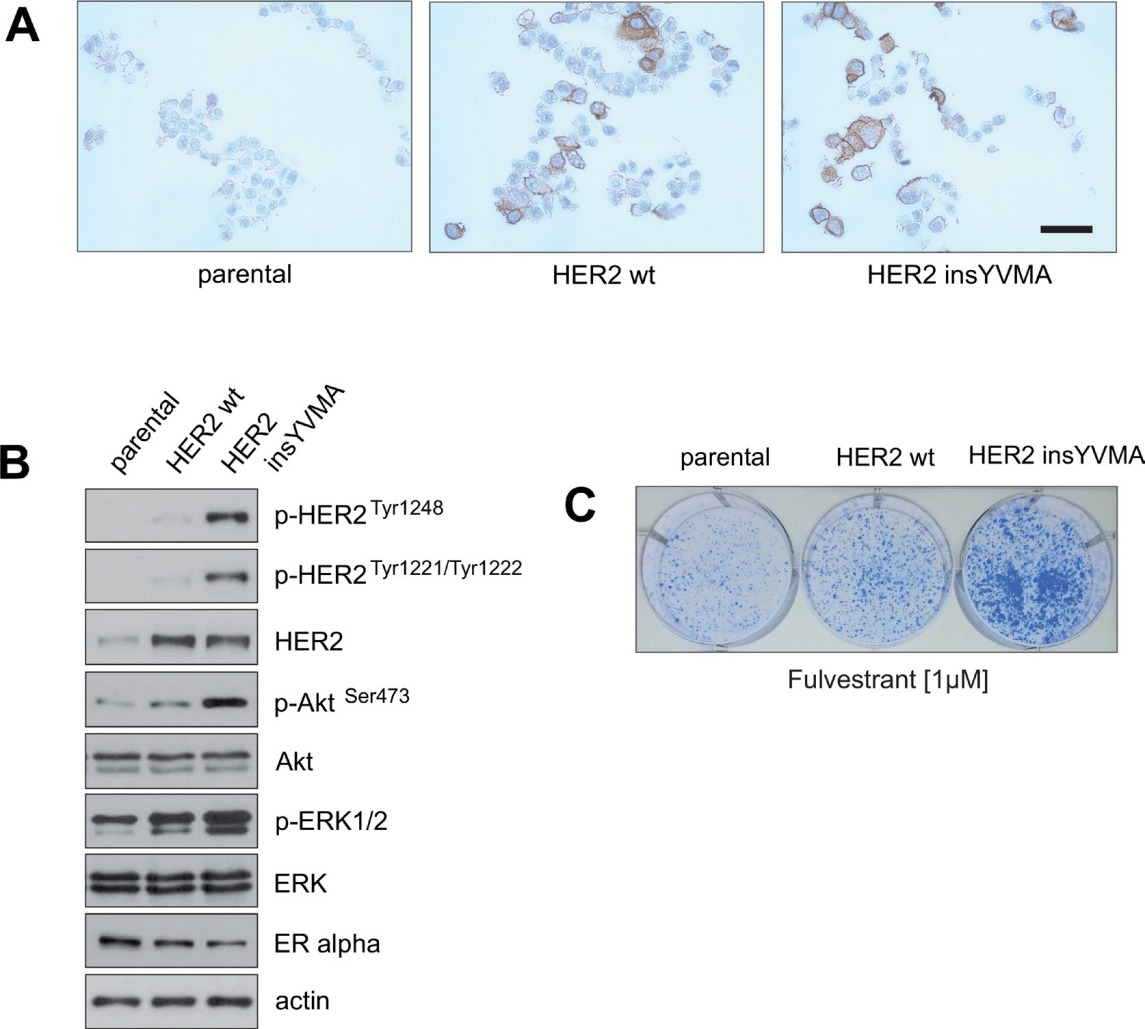
Establishment of fulvestrant-resistant ERα-positive breast cancer cells that express a constitutively active HER2 mutant that is resistant to inhibition by trastuzumab and lapatinib (**A**) MCF7 cells were retrovirally transduced to express HER2 wild-type (wt) or the constitutively active HER2 mutant insYVMA [40]. After selection, surviving cells and parental MCF7 cells were fixed and expression of HER2 was evidenced by immunohistochemical staining. Bar 50 μm. (**B**) Extracts from the indicated cells were analyzed by immunoblotting with the indicated antibodies. (**C**) Equal numbers of negative control parental and MCF7 cells stably expressing HER2 wt or HER2 insYVMA were seeded on 6-well culture plates and treated with the indicated fulvestrant concentration. After 11 days cells were fixed and stained.

The observations above show that PIs dephosphorylate HER2 and thereby render it inactive, a mode of HER2 suppression that differs from inhibitory antibodies such as trastuzumab or the tyrosine kinase inhibitors (TKIs) lapatinib and neratinib. We therefore hypothesized that PIs may be able to inhibit constitutively active HER2 mutants that render breast cancer cells resistant to commonly used anti-HER2 therapies. To investigate this hypothesis we created MCF7 cells that ectopically express either wild-type (wt), HER2 or HER2 insYVMA a constitutively active mutant that is resistant to trastuzumab and lapatinib [40]. Ectopic HER2 protein expression was monitored by immunohistochemical staining (Figure 6A).

Immunoblotting showed that the constitutively active HER2 insYVMA expressing MCF7 cells displayed markedly increased levels of p-HER2 ^Tyr1248^, p-HER2^Tyr1221/1222^, p-Akt^Ser473^ and p-ERK1/2 in comparison to HER2 wt cells, even though the HER2 insYVMA cells expressed slightly lower amounts of HER2 protein (Figure 6B). Notably, MCF7 HER2 insYVMA and HER2 wt displayed reduced levels of ERα in comparison to the negative control parental cells (Figure 6B), consistant with observations made previously by others [41, 42].

To determine whether the HER2-overexpressing MCF7 cells display intrinsic resistance to drugs that target ERα, equal numbers of negative control parental, HER2 wt and HER2 insYVMA cells were cultured in the presence of fulvestrant as indicated (Figure 6C). After 10 days, MCF7 HER2 insYVMA displayed the highest colony outgrowth in the presence of fulvestrant. Interestingly, MCF7 HER2 wt cells showed only a slightly increased outgrowth in comparison to negative control cells, but a much more reduced outgrowth in comparison to MCF7 HER2 insYVMA cells (Figure 6C).

These observation show that increased expression of HER2 wt only moderately increases intrinsic resistance of ER+/HER2+ breast cancer cells to endocrine therapy, supporting the notion that intrinsic resistance to ERα-targeting drugs in ER+/HER2+ breast cancer patients might often be driven by HER2 mutants.

### Neither HER2 wt nor constitutively active HER2 insYVMA can protect ER+ breast cancer cells from carfilzomib or bortezomib-induced cell death

**Figure 7:**
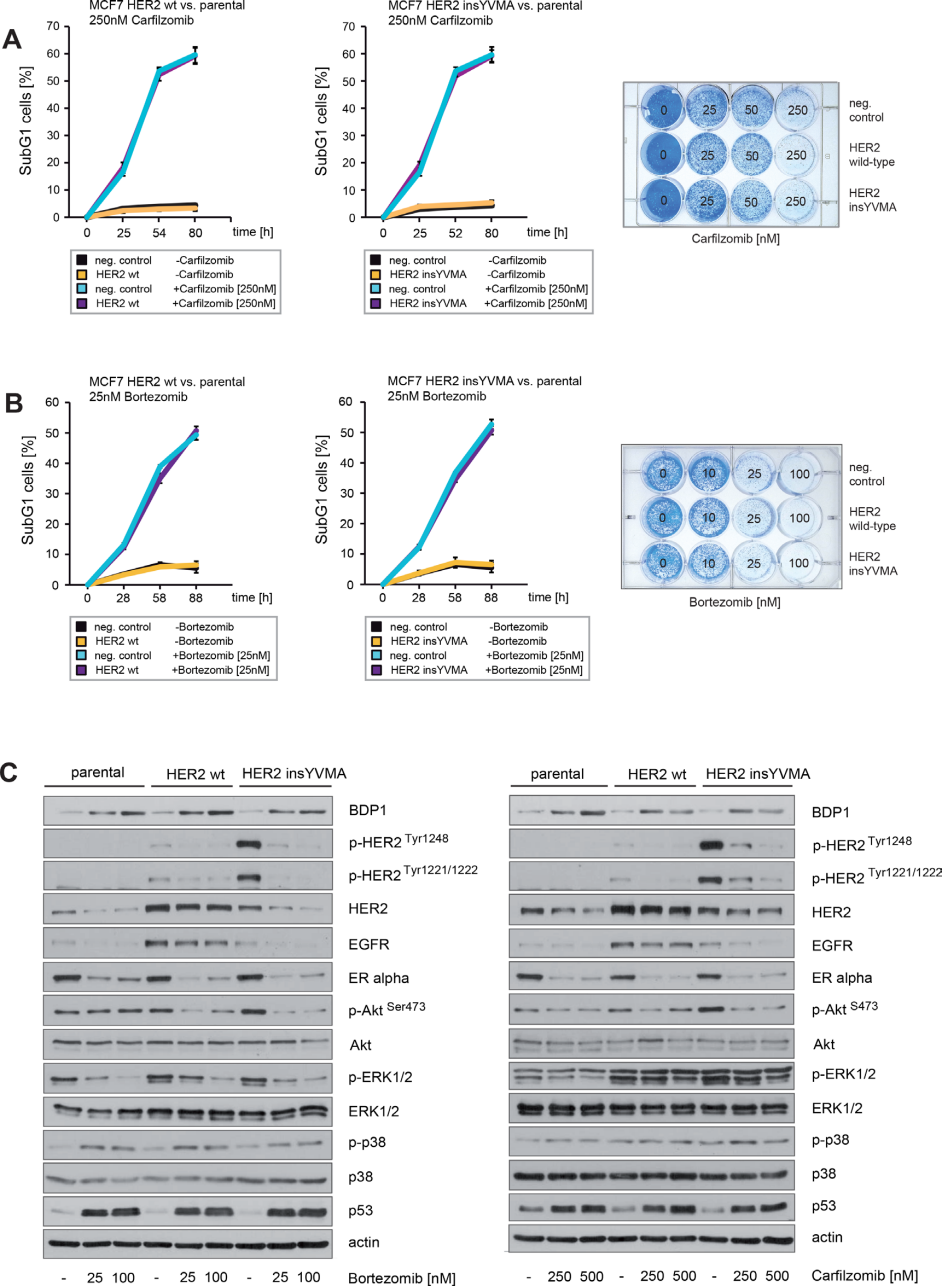
Neither enforced expression of HER2 wt nor constitutively active HER2 insYVMA can protect ER+ breast cancer cells from carfilzomib or bortezomib induced cell death (**A**) Carfilzomib and (**B**) bortezomib induce cell death in a time-dependent manner. Parental, HER2 wt or HER2 insYVMA MCF7 cells were cultured in the presence or absence of the indicated carfilzomib or bortezomib concentrations. The percentage of SubG1 cells was evaluated at different time points using propidium iodide staining and flow cytometry (left and middle panel). Mean values ± s.d. of three independent experiments are presented. Equal numbers of HER2 wt, HER2 insYVMA and negative control parental MCF7 cells were seeded on 12-well culture plates and treated with the indicated carfilzomib or bortezomib concentrations (right panels). After 9 days cells were fixed and stained. (**C**) MCF7 cells stably expressing HER2 wt or HER2 insYVMA and negative parental control cells were cultured with the indicated carfilzomib and bortezomib concentrations for 32 hours. Western blots of protein lysates were probed with the indicated antibodies.

To examine whether PIs can act against ERα+/HER2+ breast cancer cells that express a constitutively active HER2 mutant that confers resistance to trastuzumab and lapatinib and intrinsic resistance to endocrine therapy, MCF7 HER2 insYVMA [40], MCF7 HER2 wt [40] and parental MCF7 cells were cultured in the presence of various carfilzomib or bortezomib concentrations as indicated. Induction of cell death upon carfilzomib and bortezomib treatment was assessed by quantification of SubG1 cells (Figure 7A, 7B, left+middle panel) and by colony-forming assays (Figure 7A, 7B, right panel). Notably, all cells were killed after 9 days when cultured in the presence of 250 nM carfilzomib (Figure 7A, right panel) or 25 nM bortezomib (Figure 7B, right panel).

Immunoblotting demonstrated that both carfilzomib and bortezomib increased levels of BDP1 (Figure 7C). At the same time we observed markedly decreased HER2 phosphorylation and inhibition of the downstream pathways PI3K/Akt and Ras/MAPK, as indicated by decreased levels of p-HER2^Tyr1248^, p-HER2^Tyr1221/1222^, p-Akt^Ser473^ and p-ERK1/2 (Figure 7C). In the experiments above we also detected markedly decreased expression of ERα in response to carfilzomib and bortezomib (Figure 2B, 2D). These observations therefore indicate that carfilzomib and bortezomib efficiently inhibit the activity of both HER2 and ERα. In addition, both proteasome inhibitors strongly decreased EGFR and led to increased phosphorylation of p38 (Figure 7C).

Collectively, these results show that PIs potently decrease HER2 phosphorylation and activity, thereby attenuating downstream signaling pathways such as PI3K/Akt and Ras/MAPK that confer endocrine therapy resistance and estrogen-independence in ER+ breast cancer [18, 19]. In addition, PIs also induce dephosphorylation and inactivation of constitutively active HER2 that is resistant to trastuzumab and lapatinib. PIs might therefore be a potent treatment option for ERα+/HER2+ breast tumors and breast cancer patients with mutated HER2.

### Carfilzomib and bortezomib act in concert with lapatinib to inhibit HER2 activity and to induce cell death in ER+/HER2+ breast cancer cells

PIs stabilize BDP1 that dephosphorylates HER2 and thereby revert it into an inactive state, while commonly used anti-HER2 agents exert their effects through other mechanisms (Figure 4C). We therefore investigated next whether PIs and lapatinib can cooperate to inhibit HER2 activity.

**Figure 8:**
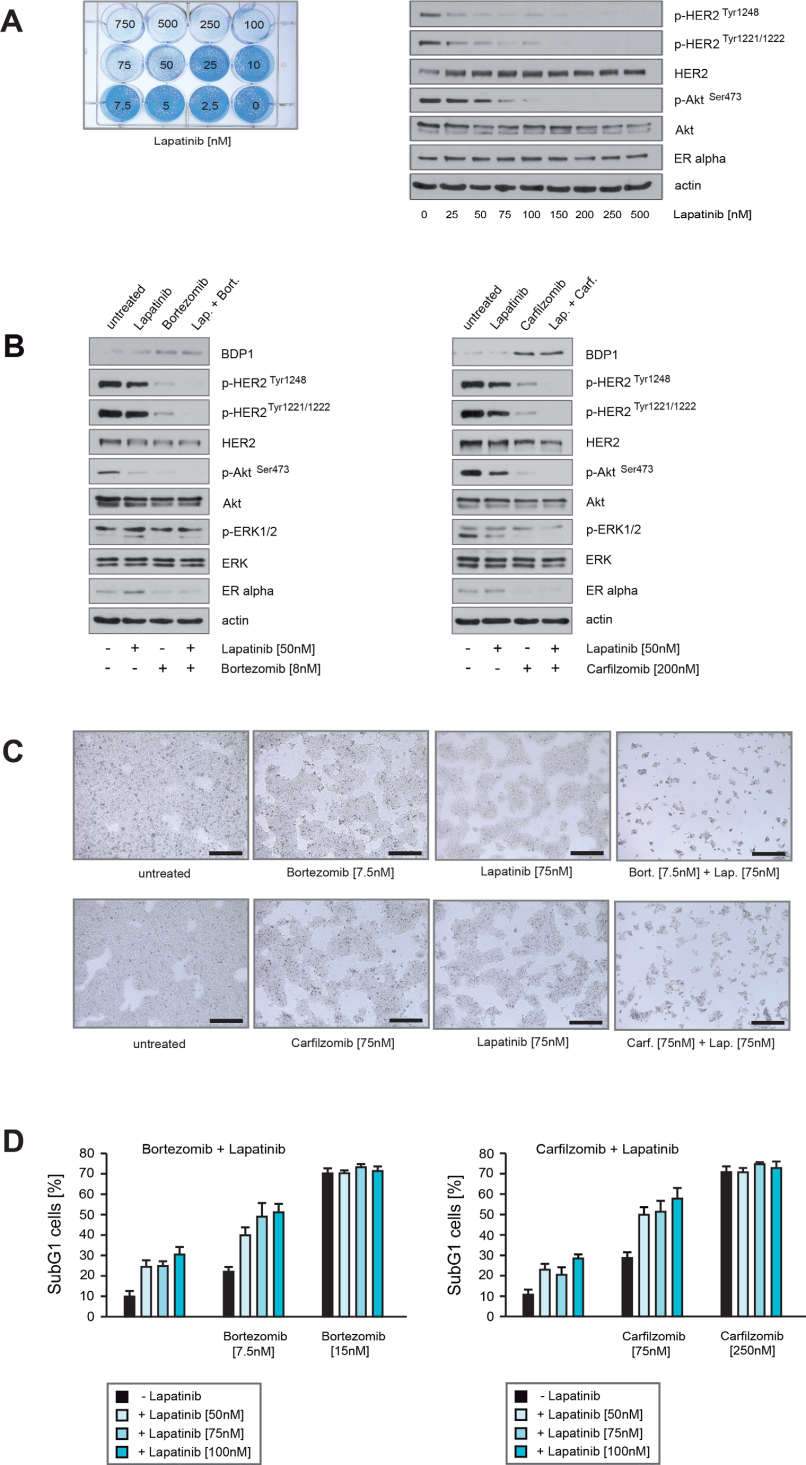
Carfilzomib and bortezomib act in concert with lapatinib to inhibit HER2 activity and to induce cell death in ER+/HER2+ breast cancer cells (**A**) Equal numbers of BT474 cells were seeded on 12-well culture plates and treated with the indicated lapatinib concentrations. After 10 days cells were fixed and stained (left panel). BT474 cells were cultured in the presence of the indicated lapatinib concentrations for 32 hours. Western blots of protein lysates were probed with the indicated antibodies (right panel). (**B**) BT474 cells were cultured in the absence or in the presence of the indicated concentrations of lapatinib, bortezomib or both drugs together (left panel) or BT474 cells were cultured in the absence or presence of the indicated concentrations of lapatinib, carfilzomib or in combination (right panel). After 30 hours cells were harvested. Western blots of the protein lysates were probed with the indicated antibodies. (**C**) Equal numbers of BT474 cells were seeded on 6-well culture plates and cultured either with or without the indicated lapatinib, bortezomib or carfilzomib concentrations. After 12 days cells were fixed. Macroscopic photos are shown of the fixed cell colonies for BT474 cells treated with lapatinib, bortezomib and lapatinib plus bortezomib (upper panel) and lapatinib, carfilzomib and lapatinib plus carfilzomib (lower panel). Bar 100 μm. (**D**) To determine the induction of cell death relative to the applied lapatinib and or bortezomib/carfilzomib concentrations equal numbers of BT474 cells were seeded on 12-well culture plates and cultured in the absence or in the presence of the indicated drug concentrations for 5 days. The percentage of SubG1 cells was evaluated using propidium iodide staining and flow cytometry. Mean values ± s.d. of three independent experiments are presented.

First we determined which concentrations of lapatinib are needed to induce growth arrest in BT474 cells as assessed by colony-forming assays (Figure 8A, left panel). Lapatinib concentrations ≥ 50 nM markedly reduced colony outgrowth (Figure 8A, left panel). Immunoblotting demonstrated that lapatinib concentrations ≥ 50 nM suppressed HER2 autophosphorylation and inhibited Akt, as indicated by decreased levels of p-HER2^Tyr1248^, p-HER2^Tyr1221/1222^ and p-Akt^Ser473^ (Figure 8A, right panel).

To address the question whether PIs and lapatinib cooperate to inhibit HER2 activity, BT474 cells were cultured in the presence of sublethal concentrations of carfilzomib, bortezomib or lapatinib either alone or in combination, and harvested at equal time points. Immunoblotting revealed that PIs combined with lapatinib led to stronger reduction of p-HER2^Tyr1248^, p-HER2^Tyr1221/1222^ and p-Akt^Ser473^ than did the PIs or lapatinib alone (Figure 8B). Notably, BT474 cells treated with PIs exhibited increased levels of BDP1 and decreased levels of ERα (Figure 8B). Next, equal numbers of BT474 cells were cultured in the presence of carfilzomib, bortezomib or lapatinib for 12 days, either alone or in combination. Lapatinib plus bortezomib (Figure 8C, upper panel) and lapatinib plus carfilzomib (Figure 8C, lower panel) caused a stronger decrease in colony outgrowth than if the drugs were applied alone. Induction of cell death upon lapatinib, carfilzomib and bortezomib treatment was assessed by quantification of SubG1 cells (Figure 8D). The number of SubG1 cells detected after sublethal treatment either with carfilzomib plus lapatinib or bortezomib plus lapatinib nearly corresponds to the sum of SubG1 cells that were detected when these drugs were administrated alone.

### Carfilzomib and bortezomib cause cell death in ER+/HER2-amplified breast cancer cells with acquired resistance to lapatinib

**Figure 9:**
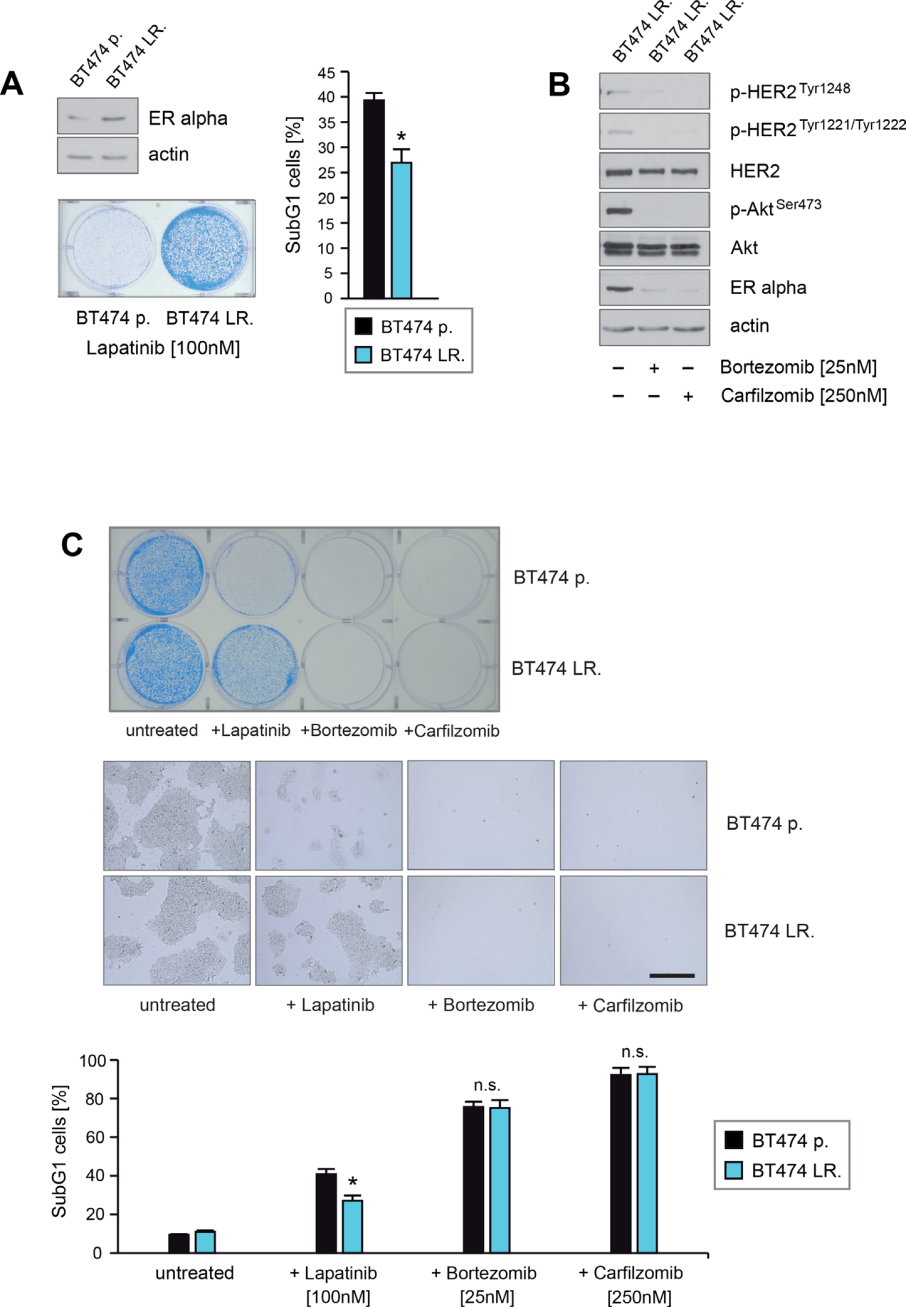
Carfilzomib and bortezomib induce cell death in ER+/HER2-amplified breast cancer cells that have acquired resistance to lapatinib (**A**) BT474 cells were cultured in the absence or presence of lapatinib concentrations > 75 nM for 9 weeks and 15 passages. Untreated parental (BT474 p.) and BT474 cells which survived and continued to grow in the presence of lapatinib (BT474 LR.) were harvested. Protein lysates were assayed by immunoblotting for the level of ERα expression. β-actin served as loading control (left upper panel). Equal amounts (5 × 10^3^) of BT474 p. and BT474 LR. cells were seeded per well on 6-well culture plates and cultured in the presence of 100 nM lapatinib. After 12 days cells were either fixed and stained (left lower panel) or cells were harvested after 5 days to determine induction of cell death. The percentage of SubG1 cells was evaluated using propidium iodide staining and flow cytometry. Mean values ± s.d. of three independent experiments are presented (right panel). *P*-values < 0.05 are indicated by asterisks. (**B**) BT474 LR. cells were cultured in presence of the indicated lapatinib, bortezomib or carfilzomib concentrations. Western blots of protein lysates were probed with the indicated antibodies. (**C**) Equal amounts of BT474 p. and BT474 LR. cells were seeded on 6-well culture plates and cultured either with or without the indicated concentrations of lapatinib, bortezomib or carfilzomib, respectively. For colony forming assay the cells were fixed and stained after 12 days cells (upper panel). Macroscopic photos of the fixed cell colonies are shown in the middle panel. Bar 100 mm (middle panel). To determine the induction of cell death cells were harvested after 5 days. The percentage of SubG1 cells was evaluated using propidium iodide staining and flow cytometry. Mean values ± s.d. of four independent experiments are presented (lower panel). *P*-values < 0.05 are indicated by asterisks.

To determine whether PIs have the potential to inhibit outgrowth or to induce cell death in ER+/HER2+ breast cancer cells with acquired resistance to lapatinib, we first developed lapatinib-resistant BT474 cells by culturing them in the presence of lapatinib for 10–12 weeks. Consistant with previous reports [15–17] immunoblotting showed that BT474 cells cultured in the permanent presence of lapatinib (referred here as BT474 LR.) displayed increased expression of ERα in comparison to their non-treated counterparts (BT474 p.) (Figure 9A, upper left panel). Induction of cell death upon lapatinib treatment was assessed by quantification of SubG1 cells (Figure 9A, right panel) and by colony-forming assays (Figure 9A, left lower panel). After 12 days of lapatinib treatment significant differences in the numbers and outgrowth of colonies between BT474 LR. and BT474 p. cells were observed (Figure 9A, left lower panel) and BT474 LR. cells formed colonies even in the presence of lapatinib. Furthermore BT474 LR. cells exhibited around 25–35% less cell death in response to lapatinib than BT474 p. (Figure 9A, right panel).

**Figure 10:**
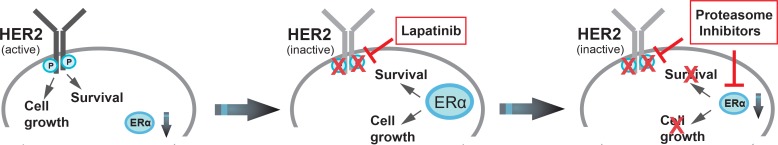
Schematic model of how proteasome inhibitors disrupt bi-directional HER2/ERα cross-talk signaling pathways in lapatinib-resistant ER+/HER2+ breast cancer cells Schematic overview of how PIs might counteract mechanisms that lead to lapatinib resistance in ERα+/HER2+ breast cancer cells, based on the results of this report and the literature. Continued activation of HER2 has a causal role in tumorigenesis, lead to tumor progression [1]. Aberrant signaling through HER2 and other members of the HER-family mediates endocrine resistance in ERα positive breast cancer [18, 19], while preclinical and clinical studies revealed that ERα co-expression attenuates the efficiency of anti-HER2 therapies [8, 9, 12, 13, 14]. Previously, published reports and our own observations demonstrate that in ERα and HER2-positive breast cancer cells that are initially sensitive to lapatinib, increased expression of ERα and enhanced ERα signaling is evident upon acquisition of resistance to lapatinib [15]. Thus, up-regulation of ERα expression and/or activity can function as an escape mechanism that leads to resistance to anti-HER2 therapy in HER2+/ER+ breast cancer patients. Thus, in HER2+/ER+ breast cancer cells, either ERα or HER2 can promote proliferation and survival. These studies and our observations demonstrate that concomitant inhibition of both HER2 and ERα is needed to achieve the best antitumor activity in ER+/HER2+ breast cancer. Our experiments demonstrate that carfilzomib and bortezomib strongly reduce phosphorylation and activation of HER2 most likely through protecting HER2-specific PEST protein tyrosine phosphatases such as BDP1 from proteasomal destruction. Additionally, both PIs strongly reduce expression of ERα protein expression. Thus, PIs might be effective in the treatment of HER2+/ERα+ breast cancer by disrupting bi-directional HER2/ERα cross-talk signaling pathways.

Next we investigated the potential of PIs to down-regulate ERα expression and to inhibit HER2 activation in BT474 LR. cells. For this purpose BT474 LR. cells were cultured either in the absence or presence of PIs as indicated, and lysed at equal time points. Immunoblotting showed that carfilzomib and bortezomib strongly reduced ERα expression and caused inhibition of HER2 and its downstream target Akt, as indicated by decreased levels of p-HER2^Tyr1248^, p-HER2^Tyr1221/1222^ and p-Akt^Ser473^ (Figure 9B). Induction of cell death upon lapatinib, carfilzomib or bortezomib treatment was assessed by colony-forming assays (Figure 9C upper+middle panel) and quantification of SubG1 cells (Figure 9C lower panel). Notably, no surviving cells could be detected when cultured in the presence of 250 nM carfilzomib or 25 nM bortezomib (Figure 9C). Thus, these findings demonstrate that carfilzomib and bortezomib are able to disrupt HER2/ERα cross-talk and to induce cell death in HER2+/ER+ breast cancer cells with aquired resistance to lapatinib. A model of how PIs might interfere with bi-directional HER2/ERα signaling pathways that lead to lapatinib resistance is illustrated in Figure 10.

### PIs decrease ERα levels and inhibit HER2 phosphorylation comparably to fulvestrant plus lapatinib, however additionally induce caspase and PARP1 cleavage

The results of this study suggest that PIs efficiently inhibit ERα/HER2 cross-talk pathways through blocking ERα expression, as well as through inhibiting HER2 activation via stabilization of BDP1, which leads to the death of ER+/HER2+ breast cancer cells. Thus PIs might be useful for clinical application.

We therefore addressed the question of whether PIs have comparable or even stronger abilities to block ERα/HER2 cross-talk pathways than drugs commonly used to treat breast cancer. To this end BT474 were cultured in the presence of lapatinib at concentrations that suppressed HER2 autophosphorylation and inhibited Akt (Figure 8A, right panel) together with combinations of fulvestrant, bortezomib and carfilzomib as indicated. After treatment all cells were harvested at equal time points. Notably, fulvestrant was given 4 hours before PIs and lapatinib to ensure that ERα is first degraded by the proteasome.

**Figure 11:**
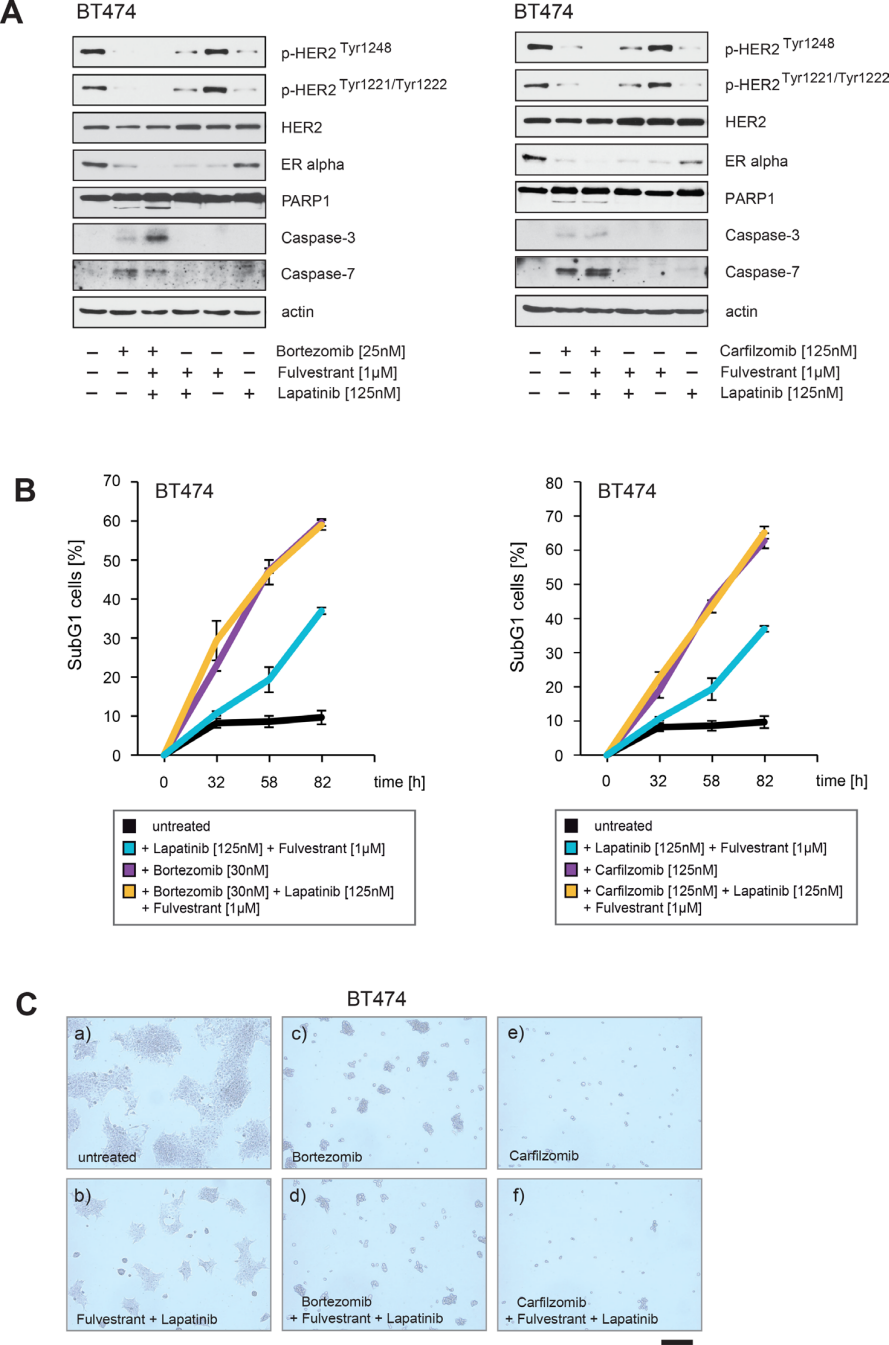
PIs decrease ERα levels and inhibit HER2 autophosphorylation to a similar extent as fulvestrant plus lapatinib, but PIs additionally lead to PARP1 and caspase cleavage (**A**) BT474 cells were cultured in the absence or in the presence of the indicated concentrations and combinations of lapatinib, fulvestrant, carfilzomib and bortezomib. Where fulvestrant was used, it was administrated 4 hours before treatment with other drugs. After 30 hours cells were harvested. Western blots of the protein lysates were probed with the indicated antibodies. (**B**) To determine the induction of cell death in response to the indicated drug combinations equal numbers of BT474 cells were seeded on 12-well culture plates and cultured in the absence or in the presence of the indicated drug concentrations. The percentage of SubG1 cells was evaluated at different time points using propidium iodide staining and flow cytometry. Mean values ± s.d. of three independent experiments are presented. (**C**) Equal numbers of BT474 cells were seeded on 6-well culture plates and cultured either with or without the indicated drug combinations. After 8 days cells were fixed. Macroscopic photos are shown of the fixed cell colonies for BT474 cells either (a) untreated or treated with (b) lapatinib [125 nM] plus fulvestrant [1 μM], (c) bortezomib [30 nM], (d) bortezomib [30 nM] plus lapatinib [125 nM] plus fulvestrant [1 μM] or (e) carfilzomib [125 nM] and (f) carfilzomib [125 nM] plus lapatinib [125 nM] plus fulvestrant [1 μM]. Bar 100 μm.

Immunoblotting revealed that PIs alone reduced HER2 phosphorylation to a similar or even stronger extent than did lapatinib, as indicated by p-HER2^Tyr1248^ and p-HER2^Tyr1221/1222^ (Figure 11A). PIs also reduced ERα levels to a comparable extent compared to fulvestrant (Figure 11A). However BT474 cells treated with PIs plus fulvestrant resulted in a stronger decrease in ERα levels than when fulvestrant and PIs were applied separately (Figure 11A). After 24 hours caspase and PARP1 cleavage appeared only in BT474 cells treated with PIs (Figure 11A). The number of SubG1 cells detected after treatment with either PIs alone or in combination with lapatinib plus fulvestrant were nearly equivalent whereas treatment with fulvestrant plus lapatinib alone resulted in a later induction and lower number of SubG1 cells (Figure 11B). Consistently in colony-forming assays, bortezomib and carfilzomib caused a stronger decrease in colony outgrowth than fulvestrant plus lapatinib and combining fulvestrant plus lapatinib treatment with bortezomib and carfilzomib had an equivalent effect in these assays as bortezomib and carfilzomib administrated alone (Figure 11C).

Collectively these data suggest that PIs have superior abilities to inhibit ERα/HER2 cross-talk pathways and thereby to suppress the growth of breast cancer cells compared to fulvestrant plus lapatinib.

## DISCUSSION

In ER+/HER2+ breast tumors, ERα and HER2 synergize to allow breast cancer cells to escape from both anti-ERα and anti-HER2 targeted therapies. Thus, ERα circumvents the effects of anti-HER2 drugs and chemotherapy whereas HER2 signaling causes endocrine resistance.

Here we report that carfilzomib and bortezomib markedly inhibit bi-directional HER2/ERα signaling pathways in HER2+/ER+ breast cancer cells. Both PIs suppress ERα expression, inhibit HER2 activity and subsequently suppress the HER2 downstream pathway PI3K/Akt. HER2 and increased activation of the PI3K/Akt pathway are major executors of endocrine resistance [18, 19]. Notably, we found that combined treatment of the ER+/HER2+ breast cancer cell line BT474 with fulvestrant plus lapatinib was not as efficient as PIs in inducing cell death (Figure 11A–11C). Furthermore, we observed that both PIs stabilize the HER2-specific tyrosine phosphatase BDP1 (Figure 2B, 2D), which is required for PI-mediated inhibition of HER2 and Akt activation as well as increased sensitivity to PI-induced cell death (Figure 5B–5D). Thereby, PIs suppress the activity of even a constitutively active HER2 variant that causes resistance to trastuzumab and lapatinib (Figure 7C).

These findings clearly demonstrate that PIs disrupt the cross-talk between HER2 and ERα signaling pathways, through mechanisms that differ from current therapeutic regimens and therefore might expand treatment opportunities for ER+/HER2+ and possibly also for other groups of breast cancer patients by breaking therapy resistance (Figure 4C and Figure 11).

Our data suggest that patients with resistance to HER2-targeted therapies also should benefit from PI treatment independent of their HR status. Futhermore, carfilzomib and bortezomib markedly decrease the phosphorylation status of a constitutively active HER2 mutant that is resistant to trastuzumab and lapatinib [40], suggesting that proteasome inhibitors could be an interesting therapeutic opportunity for breast cancer patients with mutated HER2 variants that are resistant to commonly used anti-HER2 drugs. HER2 somatic mutations represent an alternative mechanism to activate HER2 breast cancer [43]. The majority of HER2 somatic mutations in breast cancer patients are activating mutations that likely drive tumorigenesis with an overall HER2 mutation rate of approximately 1.6% in breast cancers without HER2 amplification [43]. The frequency of somatic HER2 mutations is considerably higher in patients who are refractory to anti-HER2 therapies. For example, HER2 mutations were found in six of 36 patients (16.7%) refractory to anti-HER2 therapies [44]. The TKI neratinib is actually under investigation for patients with mutated HER2 and was shown to inhibit several HER2 mutations with resistance to trastuzumab an/or lapatinib in cell culture experiments [40, 43].

We predict that PIs might have advantages over TKIs, because the mechanisms that lead to resistance to HER2-targeted therapies such as up-regulation of other growth factors or HER2/ERα cross-talk signalling pathways are also likely to attenuate the efficiency of new TKIs that target HER2 mutations. PIs on the other hand affect HER2+ breast cancer cells through multiple mechanisms and counteract HER2 activation through different mechanisms compared to TKIs for example through stabilizing the PEST-type protein tyrosine phosphatase BDP1 as we show here (Figure 4C, Figure 5). It is therefore conceivable that a stronger suppression of HER2 could possibly be achieved if both drugs are combined.

Several *in vitro* studies and clinical trials strongly suggest that ERα co-expression attenuates the efficiency of anti-HER2 drugs such as trastuzumab and lapatinib [45]. However, a variety of other mechanisms leading to intrinsic or aquired resistance to HER2-targeting therapies that are independent of ERα status have been observed among HER2+ breast cancer patients [46], including low PTEN expression, oncogenic PI3CA mutations [47], up-regulation of growth factor receptors such as other HER-family members, IGF-R1 [48], EphA2 [49], Met [50] and Axl [51]. Thus, in ongoing studies we are focusing on whether PIs also inhibits activation of other growth factor receptors like IGF-R1, FGFR1, FGFR2, Met and Axl that have been implicated in resistance to lapatinib [39] and which might be also associated with resistance to neratinib.

Our data indicate that PIs should also therapeutically target endocrine-resistant ER+/HER2- breast cancer. Frequent causes for intrinsic or aquired endocrine resistance in ER+/HER2- breast cancer include increased growth factor receptor signaling that results in ligand-independent ERα activation, genomic alterations involving the ERα gene *ESR1* such as *ESR1* gene re-arrangements with other genes, *ESR1* amplifications and *ESR1* recurrent point mutations [19]. Recurrent ERα mutations, ERα fusion proteins or postranscriptional modifications of ERα can circumvent the binding of inhibitory drugs to ERα or lead to constitutively active/ligand-independent ERα proteins thereby making breast cancer cells resistant to drugs such as SERMs (e.g. tamoxifen) and AIs. Carfilzomib and bortezomib strongly suppress ERα protein expression and markedly reduce ERα levels [32]. We assume that PIs will have advantages over therapeutic regimens that simply affect ERα activity, such as aromatase inhibitors (AIs), SERMs and SERDs in patients with ERα mutations or with deregulated growth factor receptor signaling due to HER2-amplification or increased expression of other growth factors. In these patients drugs that solely target ERα activity cause activation of survival mechanisms and must therefore be combined with a second drug to suppress rescue pathways such as PI3K/Akt/mTOR and thereby to reverse or to avoid endocrine resistance. In contrast, PIs block ERα expression and concomitantly suppress survival pathways such as PI3K/Akt. Together our observations suggest that PIs might be a therapeutic option for endocrine resistant ER+ breast cancer patients, independently of whether endocrine resistance is mediated by deregulated growth factor receptor signaling or by ERα mutations.

The results from a recently reported randomized phase II trial are consistant with our suggestion that PIs can overcome endocrine therapy resistance. In this study, AI-resistant HR+ metastatic breast cancer patients were treated with fulvestrant alone or in combination with bortezomib [52]. This trial demonstrated a significantly reduced rate of disease progression and significantly improved 12 month progression-free survival (PFS) rates in the fulvestrant plus bortezomib treated patients compared to those being treated with fulvestrant alone [52]. This trial was based on *in vitro* studies showing that fulvestrant promotes the accumulation of toxic ER aggregates in the cytoplasm that are normally cleared by the proteasome. Inhibition of the proteasome by combining fulvestrant with bortezomib was reasoned to enhance toxicity by increasing the amount of ER aggregates [53]. However the exact mechanisms by which bortezomib affects ER+ breast cancer cells within patients are unknown.

In contrast to a previously published report [53], we found that treatment of BT474 cells with PIs and fulvestrant caused a stronger reduction of ERα levels than if fulvestrant or PIs were administered alone (Figure 11A). A possible explanation for this discrepancy might be that the chronological order in which fulvestrant and PIs are added to cells determines the effect upon ERα expression.

We administered fulvestrant 4 hours before the PIs were added. It is therefore conceivable that ERα was first degraded by the proteasome, and after PIs were added the ERα expression was blocked and thereby no new ERα could further be produced. Interestingly, in the clinical trial to compare the efficiency of fulvestrant alone or in combination with bortezomib, patients in the fulvestrant + bortezomib arm received fulvestrant before bortezomib was administered [52]. Our results therefore suggest that the successful outcome of this clinical trial was probably due to a stronger suppression of ERα expression in the fulvestrant + bortezomib arm rather than through accumulation of toxic ER aggregates. Thus, further clinical studies are required to gain more information about how PIs exactly act on breast cancer cells within patients. These trials should give insights into which patients would have benefit from PIs and which combinations of PIs with other drugs might be useful. Furthermore the chronological order in which PIs are administered with other drugs might have a decisive influence on the success or failure of treatment.

Treatment of patients with bortezomib has often been limited due to high toxicity. However, clinical trials performed with carfilzomib in multiple myeloma patients showed a more favourable side effect profile compared to bortezomib [33]. Our results suggest that carfilzomib might have the potential to improve treatment opportunities for a variety of breast cancer patients. As a logical next step, an early phase clinical trial of carfilzomib in advanced breast cancer is required.

## MATERIALS AND METHODS

### Plasmids, shRNAs, reagents and antibodies

The plasmids pBabe puro-ERBB2 (Addgene Plasmid # 40978) [40], pBabe puro-ERRB2 A775_G776insYVMA (Addgene Plasmid # 40982) [40] and pBabe puro-gateway (Addgene Plasmid # 51070) [35] were gifts from Matthew Meyerson. Carfilzomib was purchased from Amgen, Thousand Oaks, CA, USA. Bortezomib was purchased from Janssen Cilag, Neuss, Germany. Lapatinib-Ditosylat was purchased from Selleckchem, Munich, Germany, Fulvestrant was purchased from Sigma Aldrich, Taufkrichen, Germany. Antibodies for detection of ERα (D-12): sc-8005, β-actin (C-4): sc-47778, Akt (H-136): sc-8312, ERK1 (C-16): sc-93, p38 (C-20): sc-535, BDP1 (B-6): sc-515058, p53 (DO-1): sc-126, EGFR (1005): sc-03 and Caspase-3 (E-8): sc-7272 were purchased from Santa Cruz Biotechnology, Heidelberg, Germany. Antibodies for detection of HER2 (#2242), p-HER2^Tyr1248^ (#2247), p-HER2^Tyr1221/1222^ (#2243), p-Akt^S473^ (#9271), p-ERK1/2 (#9106), p-p38 (#4511), PARP1 (#9532) and Caspase-7 (#9492) were purchased from Cell Signaling Technology, Heidelberg, Germany. shRNA against human BDP1 (PTPN18) and non-targeted shRNA were purchased from Sigma Aldrich (Taufkirchen, Germany). BDP1 (PTPN18) shRNA1: NM_014369/TRCN0000003044; shRNA2: NM_014369/ TRCN0000003045; shRNA3: NM_ 014369/TRCN0000003046; shRNA4: NM_014369/ TRCN0000003046; shRNA4: NM_014369/ TRCN 0000381010; shRNA5: NM_014369/ TRCN0000382258; shRNA6: NM_014369/ TRCN0000380807; shRNA7: NM_014369/ TRCN0000381001; shRNA8: NM_014369/ TRCN0000381490; shRNA9: NM_ 014369/ TRCN0000 380581; shRNA10: NM_014369/TRCN0000380069.

### Cell culture and viral transduction

MCF7 and T47D cells were purchased from ATCC, BT474 cells were purchased from CLS Cell Line Services GmbH (Heidelberg, Germany) and MDA-MB-361 cells were purchased from Sigma Aldrich (Taufkirchen, Germany). MCF7 and T47D were maintained in RPMI supplemented with 10% fetal bovine serum (Takara Clontech, Heidelberg, Germany), 1% L-glutamine and 1% penicillin/streptomycin. BT474 cells were maintained in Advanced DMEM/F12 (Takara Clontech, Heidelberg, Germany) supplemented with 10% fetal bovine serum (Takara Clontech, Heidelberg, Germany), 1% L-glutamine, 1% penicillin/streptomycin and 50 mg/ml human insulin (Sigma Aldrich, Taufkirchen, Germany). The packaging cell line Phoenix GP was used for generation of retroviruses following standard calcium phosphate protocols. Cells were selected with puromycin. For all experiments pooled, transduced selected cell clones were used.

### Other methods

Western blot analysis, FACS analysis and clonogenic assays were performed as previously described [54].

### Immunohistological staining

Immunohistochemistry was performed by using the Hercep *t*-test (Dako, Glostrup, Denmark). Staining was performed on an immunostainer (Autostainer +; Dako, Glostrup, Denkmark) according to the manufacturer's instructions.

### Cell cycle analysis

Cell cycle analysis was performed through direct DNA staining with propidium iodide. Cells were resuspended in hypotonic fluorochrome solution (50 mg/ml propidium iodide in 0.1% sodium citrate plus 0.1% TritonX-100 (Sigma Aldrich, Taufkirchen, Germany), than placed at 4°C in the dark for 1 hr before flow cytometry analysis.

### Statistical analysis

Differences between experimental groups were assessed using the Student's *t* test (Statistical Analysis System, Release 9.3). *p* values of < 0.05 were considered significant.
